# Supplementary material for: A joint diffusion/collision model for crystal growth in pure liquid metals
Source: Nat Commun. 2024 Jul 9;15:5749. doi: 10.1038/s41467-024-50182-7 (PMC11233554; doi:10.1038/s41467-024-50182-7)
Supplement: Supplementary file 1 — Supplementary Information [file 41467_2024_50182_MOESM1_ESM.docx]

**A joint diffusion/collision model for crystal growth in pure liquid metals**

Hua Men

BCAST, Brunel University London, Uxbridge, Middlesex, UB8 3PH, UK.

1. **Structural templating mechanism**

Liquid atoms at the liquid/solid (L/S) interface have a local structure similar to the crystal and will template the lattice of the crystal surface during the growth, referred to as the structural templating mechanism. The normal stacking sequence of FCC(111) orientation is ABCABC (Supplementary Fig. 1a), and the liquid atoms at the L/S interface may attach to B or C atomic positions on the underlying solid atomic layer (assuming at A positions), as shown in Supplementary Fig. 1b. The atoms attached to C positions have a wrong templating and will move to B position with the atomic displacement, *d*^CB^, where the normal templating sequence is favoured if there is a relatively large stacking fault energy, such as in solid Al.^1^ The *d*^CB^ is 0.168 nm for the Al(111) interface. The *d*^CB^ displacement needs to overcome an energy barrier by jumping from one energy minimum (C position) to another (B position), and therefore it is a thermal activated process, behaving in a diffusion-controlled mode. On the other hand, some liquid atoms may attach to B positions directly without thermal activation during the growth, behaving in a collision-limited mode.

**Supplementary Fig. 1: Schematics of structural templating mechanism at the liquid/solid interface of FCC(111) structure**. **a** FCC(111) structure has a normal templating sequence of ABCABC, with an atomic layer spacing (*d*^111^) of 0.238 nm for Al. **b** The atoms in underlying solid layer assume to take A sequence, and liquid atoms can template B or C atomic positions. The displacement from C to B position, with a distance *d*^CB^ = 0.168 nm for Al, needs to overcome an energy barrier by jumping from one energy minimum (C position) to another (B position) in the growth.

1. **Densification at the interface**

The number of atoms per layer, *N*_L_, increases from the liquid to the solid at the Al(111), (110) and (100) interfaces either at equilibrium (Fig. 1) or during the crystal growth, and the latter is exemplified by the simulation result of the Al(111) interface at the simulation time *t* = 0.06 ns and *T* = 782 K with Potential A (embedded-atom method (EAM) potential for aluminium, developed by Zope and Mishin^2^) in Supplementary Fig. 2. This result is generally agreed with the literature,^3^ which reports that the density of the layers in front of the (111) and (110) interfaces increases about 10% upon solidification, coupled to atoms hopping from the layers away from the interface towards the layers near the interface. Although the atom number of the layers at (100) interface does not increase during the growth, the atomic layer spacing becomes smaller upon ordering of the atoms in these layers, thereby providing a faster growth mechanism.^3^ The densification of the interfacial layers during the growth could be attributed to a few percent of difference in the density between liquid and solid. As a consequence, the long-range diffusion of liquid atoms from the liquid to the solid across the interfacial region is necessary for the growth to take place, where the interface has a layered structure and the jumps of the atoms from one atomic layer to another need to overcome an energy barrier between two consecutive atomic planes.

**Supplementary Fig. 2: Variation in number density at the interface during crystal growth.** **a** The density profile, *ρ*(*z*), and number of atoms per layer, *N*_L_, are plotted as a function of the distance, *z*, for the Al(111) interface at the simulation time *t* = 0.06 ns during the simulation at *T* = 782 K (undercooling Δ*T* = 160 K) with Potential A (embedded-atom method (EAM) potential for aluminium, developed by Zope and Mishin^2^). For clarity, **b** enlarged interface region in **a**, **c** *ρ*(*z*) and **d** *N*_L_ have also been displayed separately. The *N*_L_ gradually increases across the interface from the liquid to solid during the growth. (Source data are provided as a Source Data file)

1. **Solid clusters in undercooled liquids**

The softness, *S*, is calculated with the support vector machine (SVM) classifiers of machine learning. Fig. 3a displays the *S* as a function of *z* for the Al(111) system, equilibrated at 942 K with Potential A. Almost all the atoms in the bulk solid have *S* > 0, and those in the bulk liquid have *S* < 0. At the interface, some atoms have *S* ≈ 0. The classification error penalty parameter *C* = 1 was used to label these atoms. It is noted that the solid atoms within the bottom two atomic layers and the liquid atoms in the top layers of the simulation system have large negative *S*, where the atoms in both cases are adjacent to the vacuum region. It suggests that the local-structure fingerprints can’t correctly describe the atoms at the surfaces of either liquid or solid of the simulation system. With increasing undercooling, the atoms in bulk liquid have 1 > *S* > -1 (Fig. 3b). The increase in *S* for bulk liquid indicates that some atoms have a structure similar to the crystal, consistent with the fact that both size and quantity of solid clusters in bulk liquid increase at higher undercooling.^4^

1. **Rough liquid/solid interfaces**

The L/S interface of pure metals is rough at an atomic-level at equilibrium. Supplementary Fig. 3 shows the top views of atomic arrangements of solid atoms at the Al (110) and (100) interfaces equilibrated at *T*_m_ with Potential A, where the trained SVM classifier is used to identify solid atoms. The Al(100) interface spans about 3 atomic layers (6.18 Å in width), and the Al(110) interface extends to about 5 atomic layers (7.28 Å in width). In term of the width, both is approximate to the Al(111) interface (7.14 Å in width) (Fig. 2a). It appears no substantial difference exists in the atomic-level roughness among the (111), (110) and (100) interfaces.

**Supplementary Fig. 3: Rough Al (110) and (100) interfaces.** Top views of atomic arrangements of solid atoms **a** at the Al (110) and **b** (100) interfaces equilibrated at melting point (*T*_m_) with Potentials A, where S1 (blue spheres), L1 (cyan spheres), L2 (mauve spheres), L3 (yellow spheres), L4 (orange spheres) and L5 (iceblue spheres) denote the 1^st^ solid layer to 5^th^ liquid layer, respectively, at the interface. The solid atoms are identified by the machine learning with support vector machine (SVM) classifier. At the interface, the solid atoms are usually located at the equilibrium atomic positions provided by the solid underlying layer, with a normal templating sequence.

All three interfaces remain planar during the growth at small undercooling (Supplementary Figs. 4a, 5a and 6a). However, the multiple spherical caps develop at the (100) and (110) interfaces at large undercooling (Supplementary Figs. 5b and 6b). The transition from the planar growth to the multiple spherical growth starts at the undercooling (Δ*T*) of 550 K at the (100) interface and 370 K at the (110) interface, respectively. The multiple spherical growth is attributed to the free growth of the spikes of rough L/S interfaces while the size of the spikes reaches the critical size, 2*r**, of the free growth at large undercooling. According to the criterion of free growth model:^5^ 2*r** = 4*σ*/Δ*S*Δ*T*_rg_, where *σ* is the L/S interfacial energy, Δ*S* is the entropy of fusion per unit volume and Δ*T*_rg_ is the free growth undercooling, here Δ*T*_rg_ = Δ*T*. The 2*r** decreases with increasing Δ*T* (Supplementary Fig. 7). The calculated 2*r** is 28.4 nm, 0.95 nm and 0.87 nm, respectively, at Δ*T* = 20 K, 600 K and 650 K for pure Al from the thermodynamic data available in literature.^6,7^ The cross sections of the (100) and (110) interfaces of the simulation systems in this study are 8.2 nm × 8.2 nm and 8.8 nm × 6.2 nm, respectively. Both is much smaller than the critical size (28.4 nm) at Δ*T* = 20 K, and therefore the free growth of the spikes of rough L/S interface will not start at small undercooling. However, the critical size 2*r** decreases to less than 1 nm at Δ*T* = 600 K or 650 K. The size of the spikes, which is a fraction of cross section of the interfaces, becomes comparable to the 2*r** and some spikes can start the free growth, leading to the transition from the planar growth to the multiple spherical growth.

**Supplementary Fig. 4: Crystal growth at (111) interface**. Front views of the snapshots of the Al (111) system during the simulation at **a** *T* = 927.7 K (undercooling Δ*T* = 10 K) and **b** 687.7 K (Δ*T* = 250 K) with Potential B (EAM potential for aluminium, developed by Song and Mendelev^8^). The solidified Al displays a normal templating sequence above the crossover temperature, *T*_c_, and stacking fault forms and dies out later in the growth at a temperature slightly below *T*_c_.

**Supplementary Fig. 5: Free growth of rough (100) interface at deep undercooling**. Front views of the snapshots for the Al(100) system are displayed during the simulations **a** at undercooling Δ*T* = 20 K and **b** 600 K with Potential A. The liquid/solid (L/S) interface remains planar during the growth at Δ*T* = 20 K, and exhibits the multiple spherical growth at Δ*T* = 600 K.

**Supplementary Fig. 6: Free growth of rough (110) interfaces**. Front views of the snapshots for the Al(110) system are displayed during the simulations **a** at undercooling Δ*T* = 20 K and **b** 650 K with Potential A. The liquid/solid (L/S) interface remains planar during the growth at Δ*T* = 20 K, and shows the multiple spherical growth at Δ*T* = 650 K.

**Supplementary Fig. 7: Relationship between the critical size and undercooling of free growth**. The free growth undercooling, Δ*T*_rg_, and critical size, 2*r**, for pure Al are calculated, according to the criterion of free growth model.^5^ The 2*r** is 28.4 nm, 0.95 nm and 0.87 nm, respectively, at the undercooling Δ*T* = 20 K, 600 K and 650 K. (Source data are provided as a Source Data file)

1. **Local bond-order analysis**

For the training of the machine learning, the local bond-order analysis was employed to label solid and liquid atoms, according to an order parameter, *α*, which is the fraction of crystal-like bonds for each atom with the nearest neighbours. *α* >= 0.4 for the atoms in the bulk solid and *α* < 0.1 in the bulk liquid, as shown in Supplementary Fig. 8. *α* = 0.4 is the optimal hyperparameter in the grid-search with both the SVM and neural network (NN) classifiers, and very close to the threshold of 0.5 for the FCC structure to identify solid and liquid atoms at the interface with the local bond-order analysis in our previous study.^9^ In this study, the atoms with *α* ≥ 0.4 and *α* < 0.1 are labelled as the solid and liquid, respectively. These atoms with 0.4 > *α* > 0 are labelled as the interfacial liquid atoms, and it should be pointed out that they are in the liquid status, neither partially liquid nor partially solid.^10^ The interfacial liquid atoms are subject to further investigation in order to establish the mechanism of atomic attachments at the interface during the growth.

1. **Displacement and dislocation correction mechanisms**

The crystal growth of the Al(111) interface usually takes a normal templating sequence of ABCABC of FCC structure at small undercooling (Supplementary Fig. 4a), where wrong templating can be corrected by atomic displacements from C to B positions on the underlying solid atomic layer (assuming at A positions) due to fast kinetics at the L/S interface. Fig. 9 displays the top view of a snapshot of the 1^st^ interfacial liquid (L1) layer (yellow) superimposed on the 1^st^ solid (S1) layer (blue) at *t* = 0.06 ns during the simulation at *T* = 782 K (Δ*T* = 160 K) with Potential A, where the arrows (or short lines) represent the atomic displacements from 0.06 to 0.069 ns in the L1. Some liquid atoms have the wrong templating at *t* = 0.06 ns, and nearly all the atoms initially at C positions have moved to B positions to maintain the registry to the crystal within 9 ps. This is referred to as the displacement correction mechanism, which needs to overcome an energy barrier from one local energy minimum (C position) to another (B position) and therefore is thermal activated. One expects that the displacement correction mechanism also works at high undercooling, and in some cases this mechanism fails due to the slowing interface kinetics, leading to formation of the stacking fault and twin boundaries. The stacking fault may be eliminated later by the dislocation motion, referred to as dislocation correction mechanism, which is also thermal activated. On the other hand, the growth of Al(110) and (100) interfaces always takes a normal templating sequence of ABAB of FCC structure at either small or large undercooling (Supplementary Figs. 5, 6 and 9), and as a consequence there is no contribution from either displacement or dislocation correction mechanisms to the thermal activation in the crystal growth.

**Supplementary Fig. 8: Local bond-order analysis**. The order parameter, *α*, is plotted as a function of *z* for the Al(111) system equilibrated at 942 K with Potential A. The *α* is defined as the fraction of crystal-like bonds of an atom with its nearest neighbours, calculated with the local bond-order analysis. The dashed lines are used to indicate that all the atoms in bulk solid have *α* > 0.4, and those in bulk liquid have *α* < 0.1. (Source data are provided as a Source Data file)

1. **Development of the joint collision/diffusion model**

Assuming there is *N* liquid atoms with an average thermal velocity of (3k_B_*T*/*m*)^1/2^, where k_B_ is Boltzmann’s constant, *T* is absolute temperature and *m* is the mass of the atom, in a single atomic layer at the L/S interface, the *N* atoms need to attach to the surface of the crystal to move forward the L/S interface by an atomic layer spacing of *a*.

According to the collision theory, each of the *N* liquid atoms will attach to the surface of crystal deterministically (so the probability *p*^c^ = 1), leading to the Equation (2). If the crystal growth is a diffusion-controlled process with an activation energy, *Q*, each of the *N* liquid atoms will attach to the crystal with a probability of *p*^d^:

$p^{d}=\exp\left( -\frac{Q}{k_{B}T} \right)=\exp\left( -\frac{Q}{k_{B}T} \right)^{\frac{N}{N}}$. (S1)

The Equation (2) becomes:

$V= f\frac{a}{\lambda}\sqrt{\frac{3k_{B}T}{m}}\exp\left( -\frac{Q}{k_{B}T} \right)\left( 1-\exp\left( -\frac{\Delta\mu}{k_{B}T} \right) \right)$, (S2)

where Δ*μ* is the driving force of the growth. The probability for one atom in the *N* liquid atoms to attach to the interface is:

$p^{1}=exp\left( -\frac{Q}{k_{B}T} \right)^{\frac{1}{N}}$, (S3)

and thus, the probability for all the *N* liquid atoms can be expressed as:

$$p^{N}=\exp\left( -\frac{Q}{k_{B}T} \right)^{\frac{1}{N}}\cdot\exp\left( -\frac{Q}{k_{B}T} \right)^{\frac{1}{N}}\cdot\cdot\cdot\cdot\cdot\cdot\exp\left( -\frac{Q}{k_{B}T} \right)^{\frac{1}{N}}$$

$=exp\left( -\frac{Q}{k_{B}T} \right)^{\frac{N}{N}}=\exp\left( -\frac{Q}{k_{B}T} \right)=p^{d}$. (S4)

If only *m* atoms (*m* < *N*) of the *N* liquid atoms need thermal activation, the probability for each of the *m* atoms to attach to the crystal will be:

$p^{m}=\exp\left( -\frac{Q}{k_{B}T} \right)^{\frac{m}{N}}$. (S5)

The growth of other (*N* – *m* + 1) atoms is athermal with activation energy of *Q*_c_ = 0 eV, and the probability for each of the (*N* – *m* + 1) atoms to attach to the crystal is:

$p^{N-m+1}=\exp\left( -\frac{Q_{c}}{k_{B}T} \right)^{\frac{N-m+1}{N}}=1$. (S6)

Thus, for all *N* atoms:

$p^{N}=\exp\left( -\frac{Q}{k_{B}T} \right)^{\frac{m}{N}}\cdot\exp\left( -\frac{Q_{c}}{k_{B}T} \right)^{\frac{N-m+1}{N}}=\exp\left( -\frac{Q}{k_{B}T} \right)^{\frac{m}{N}}=p^{m}$. (S7)

The growth velocity for the case that a fraction of *x*_therm_ = *m*/*N* of liquid atoms is thermal activated can be expressed as:

$V= f\frac{a}{\lambda}\sqrt{\frac{3k_{B}T}{m}}{\exp\left( -\frac{Q}{k_{B}T} \right)}^{x_{\mathrm{therm}}}\left( 1-\exp\left( -\frac{\Delta\mu}{k_{B}T} \right) \right)$. (S8)

It produces our joint collision/diffusion model in Equation (4) as a general case for the crystal growth.

1. **Fitting crystal growth data**

The simulated growth velocity *V* is calculated as a function of Δ*T* for the Al(111), (110) and (100) interfaces with Equation (9), and then fitted with Equation (4) by using the SVM regression method of machine learning with custom kernel. In Equation (4), *f* = 0.27 is adopted from the work of Broughton et al.^11^, *λ* = 0.4*a*,^11^ Δ*μ* is calculated with Pandat Al database,^12^ and *x*_therm_ = *b*Δ*T + c*, where *b* and *c* are the fitting parameters. For *x*_therm_ = 0, Equation (4) is reduced to the collision-limited model:

$V= f\frac{a}{\lambda}\sqrt{\frac{3k_{B}T}{m}}\left( 1-\exp\left( -\frac{\Delta\mu}{k_{B}T} \right) \right)$. (S9)

For *x*_therm_ = 1, Equation (4) is reduced to the diffusion-controlled model:

$V= f\frac{a}{\lambda}\sqrt{\frac{3k_{B}T}{m}}\exp\left( -\frac{Q}{k_{B}T} \right)\left( 1-\exp\left( -\frac{\Delta\mu}{k_{B}T} \right) \right)$. (S10)

**Supplementary Fig. 9: Crystal growth at (110) and (100) interfaces**. Front views of the snapshots of **a** Al (110) and **b** (100) systems during the simulations at undercooling Δ*T* = 350 K with Potential A. The solidified Al has a normal templating sequence of ABAB.

1. **Gibbs free energies of BCC and FCC W**

The Gibbs free energy, *G*_bulk_, of the BCC and FCC W, is calculated with Pandat W database,^12^ as shown in Supplementary Fig. 10. The *G*_bulk_ of FCC W becomes positive below about 550 K and that of BCC W remains negative.

**Supplementary Fig. 10: Gibbs free energy of BCC and FCC W**. The Gibbs free energy, *G*_bulk_, of the BCC and FCC W is plotted as a function of temperature, *T*. The dashed line, *G*_bulk_ = 0 kJ mol^-1^, is used to indicate that the *G*_bulk_ of FCC W becomes positive below about 550 K and that of BCC W remains negative. (Source data are provided as a Source Data file)

**Supplementary References**:

1. Hammer, B., Jacobsen, K. W., Milman, V. & Payne, M. C. Stacking fault energies in aluminium. *J. Phys.: Condens. Matter* **4**, 10453-10460 (1992).

2. Zope, R. R. & Mishin, Y. Interatomic potentials for atomistic simulations of the Ti-Al system. *Phys. Rev. B* **68**, 024102 (2003).

3. Huitema, H. E. A., Vlot, M. J. & van der Eerden, J. P. Simulations of crystal growth from Lennard-Jones melt: Detailed measurements of the interface structure. *J. Chem. Phys*. **111**, 4714-4723 (1999).

4. Kelton, K.F. Crystal nucleation in liquids and glasses. *Solid State Physics* **45**, 75-177 (1991).

5. Greer, A. L., Bunn, A. M., Tronche, A., Evans, P. V. & Bristow, D. J. Modelling of inoculation of metallic melts: application to grain refinement of aluminium by Al–Ti–B. *Acta Mater*. **48**, 2823-2835 (2000).

6. Eustathopoulos, N., Coudurier, L., Joud, J. C. & Desré, P. Tension interfaciale solide-liquide des systémes Al-Sn, Al-In et Al-Sn-In. *J. Cryst. Growth* **33**, 105-115 (1976).

7. Brandes, E. A. (ed.), *Smithells Metals Reference Book* 6th edn. (Butterworths, London, 1983) pp. 8-1-14-1.

8. Song, H. & Mendelev, M. I. Molecular dynamics study of mechanism of solid–liquid interface migration and defect formation in Al_3_Sm alloy. *JOM* **73**, 2312–2319 (2021).

9. Men, H. & Fan, Z. Prenucleation induced by crystalline substrates. *Metall. Mater. Trans. A* **49**, 2766–2777 (2018).

10. Jackson, K. A. The interface kinetics of crystal growth processes. *Interface Sci*. **10**, 159–169 (2002).

11. Broughton, J. Q., Gilmer, G. H. & Jackson, K. A. Crystallization rates of a Lennard-Jones liquid. *Phys. Rev. Lett*. **49**, 1496-1500 (1982).

12. Pandat with Pan Al Database Version 2021. Available online: www.computherm.com (accessed on 6 December 2021).
